# Regional Differences in Microbial Infiltration of Brain Tissue from Alzheimer’s Disease Patients and Control Individuals

**DOI:** 10.3390/brainsci14070677

**Published:** 2024-07-03

**Authors:** T. Bucky Jones, Ping Chu, Brooke Wilkey, Leigha Lynch, Garilyn Jentarra

**Affiliations:** 1 College of Graduate Studies, Midwestern University, Glendale, AZ 85308, USA; bjones1@midwestern.edu (T.B.J.); pchu@midwestern.edu (P.C.); llynch@midwestern.edu (L.L.); 2 Arizona College of Osteopathic Medicine, Midwestern University, Glendale, AZ 85308, USA; brookewilkey@creighton.edu; 3 School of Medicine, Creighton University, Phoenix, AZ 85012, USA

**Keywords:** microbiome, Alzheimer’s disease, LPS, 16S rRNA sequencing, brain, serum

## Abstract

Alzheimer’s disease (AD) is characterized by cognitive decline and neuropathology including amyloid beta (Aβ) plaques and neurofibrillary tangles (tau). Factors initiating or driving these pathologies remain unclear, though microbes have been increasingly implicated. Our data and others’ findings indicate that microbes may be common constituents of the brain. It is notable that Aβ and tau have antimicrobial properties, suggesting a response to microbes in the brain. We used 16S rRNA sequencing to compare major bacterial phyla in post-mortem tissues from individuals exhibiting a range of neuropathology and cognitive status in two brain regions variably affected in AD. Our data indicate that strong regional differences exist, driven in part by the varied presence of Proteobacteria and Firmicutes. We confirmed our data using ELISA of bacterial lipopolysaccharide (LPS) and lipoteichoic acid in the same brain tissue. We identified a potential association between the composition of phyla and the presence of neuropathology but not cognitive status. Declining cognition and increasing pathology correlated closely with serum LPS, but not brain levels of LPS, although brain LPS showed a strong negative correlation with cerebral amyloid angiopathy. Collectively, our data suggest a region-specific heterogeneity of microbial populations in brain tissue potentially associated with neurodegenerative pathology.

## 1. Introduction

Alzheimer’s disease (AD) is a devastating degenerative neurological disorder characterized by progressive cognitive decline [1,2]. The neuropathology associated with this disorder includes the early and increasing accumulation of amyloid beta (Aβ) plaques and neurofibrillary tangles in the brain, as well as the loss of both neurons and white matter, a pathology that is particularly prominent in the later stages of the disease. The amyloid plaques and neurofibrillary tangles can be seen to begin accumulating long before obvious cognitive dysfunction appears [3]. This extended period of disease development, over years or decades, allows ample time for intervention, which could be much more targeted if the pathology-triggering mechanisms were better understood. In recent years, an array of evidence has been produced demonstrating that both Aβ and the hyperphosphorylation of tau can be induced in response to the presence of various microbes [4,5,6,7,8]. While AD patients do not display symptoms of overt brain infections, it has been suggested that pathology may be provoked by subclinical infection or by repeated low-level exposure to microbes or their products [9]. This exposure might induce an ongoing inflammatory response consistent with what is observed in the brain with Alzheimer’s disease. Indeed, the activation of various immune cells, particularly microglia, and production of cytokines both in the central nervous system (CNS) and the periphery have been reported in AD [10,11,12,13]. Critically, it has been shown that Aβ can act as a potent antimicrobial peptide (AMP) capable of both pathogen agglutination and the formation of channels/pores in microbial target membranes [14,15,16,17]. Aβ may therefore be specifically produced in the brain to limit infection, suggesting that there may be microbes entering the brain, either deliberately or inadvertently, that are triggering an antimicrobial response. The hyperphosphorylation of tau, which drives Aβ aggregation, may also be induced by microbial infection [6,18].

Many reports have identified the presence of a range of microorganisms in brain tissue in association with AD [12,19]. These include *Chlamydia pneumoniae* [20,21,22,23,24], spirochete bacteria [25,26], herpes simplex virus 1 (HSV-1) [27,28,29,30], human herpes viruses 6A and 6B (HHV6A and HHV6B) [31,32], and fungi/yeasts including various species of *Candida* [33,34,35]. Historically, infectious diseases are most often associated with a specific causative microorganism. However, in this case, many heterogeneous microorganisms have been associated with AD, leading us to hypothesize that the identity of the microorganisms may not be as important as their chronic presence, which would be expected to drive a long-term inflammatory response. It is entirely possible, and even likely, that some microorganisms might drive a stronger defensive response than others. An individual’s own genetic background might also influence the response to the presence of microbes, tying this to the propensity for AD-associated genes to have inflammatory or immunological functions. There have been multiple reports of diverse bacterial populations detected in brain tissue via 16S rRNA gene sequencing, both in association with AD [33,36,37] and with other neurological disorders such as Parkinson’s disease and multiple sclerosis [38,39]. Studies in tissue from AD patients have identified bacterial nucleic acids in both cognitively normal control individuals and in brain tissues from AD patients, sometimes with notable differences between the bacterial populations in each [37,40]. Studies using an electronic tree of life (eToL) method using ribosomal RNA (rRNA) probes have further supported the finding that many types of microorganisms are present in the brain, even in healthy individuals [41].

The purpose of this study was to compare bacterial populations across brain regions variably affected by AD pathology in a range of individuals with varying levels of cognitive decline and pathological burden using 16S rRNA gene sequencing. Further, we sought to determine whether there was a relationship between the bacterial components identified in peripheral tissues and the patterns of microbial infiltrates in the brains of the same individuals. Our findings demonstrate that the predominance of bacterial taxa varies distinctly by brain region although this is not influenced by the level of neuropathology or cognitive status. Specifically, the superior frontal gyrus (SFG) is predominated by Gram-positive bacterial taxa while the inferior temporal gyrus (ITG) predominantly contains Gram-negative taxa. In addition, we found that serum lipopolysaccharide (LPS) levels were elevated in AD and high-pathology, cognitively normal (HPC) subjects and especially in females compared to males. We also found that elevated serum LPS levels from the same individuals correlated with greater levels of cognitive decline and higher amyloid and tau pathology, but there was no significant relationship between the levels of LPS in peripheral tissues and those found in either brain region.

## 2. Materials and Methods

### 2.1. Post-Mortem Tissues

Frozen tissue samples for these experiments were acquired from the Arizona Study of Aging and Neurodegenerative Disorders/Brain and Body Donation Program at the Banner Sun Health Research Institute (BSHRI) in Sun City, AZ, USA. At BSHRI, samples are collected under an approved institutional review board (IRB) protocol (WIRB #20120821). This protocol provides broad consent for the use of the obtained tissues. Matched SFG, ITG, serum, liver, and spleen samples were obtained from each subject. Subjects were assigned to one of four experimental groups based on cognitive status assessments prior to death and post-mortem assessments of brain pathology after death. The National Institute on Aging (NIA)’s criteria for neuropathological assessment of AD were used to guide the grouping of subjects [42]. The subject groups included non-demented normal control subjects (NDN) who did not meet criteria for AD pathology, HPC subjects who were cognitively normal but displayed intermediate levels of brain pathology consistent with AD, individuals with non-specific mild cognitive impairment (MCI) and intermediate levels of brain pathology consistent with AD, and subjects with dementia who met the neuropathology criteria for AD. Male and female subjects were included in all groups. BSHRI provided clinical characteristics for the subjects in this study. These included age, sex, and post-mortem interval (PMI) data, as well as apolipoprotein E (APOE) genotypes, Mini-Mental State Examination (MMSE) scores, and Braak scores. Additional detailed measures of brain pathology were provided and included both frontal and temporal assessments and compiled data from multiple brain regions. These measures assessed amyloid plaques, neurofibrillary tangles, and cerebral amyloid angiopathy (CAA). As assessments of microbial components were specifically being made in the experiments in this study, BSHRI also provided details pertaining to evidence of potential infectious processes in the study subjects based on clinical and post-mortem assessments.

### 2.2. DNA Extraction for Microbial DNA Sequencing

Frozen, unfixed post-mortem SGF and ITG tissue samples for each subject were processed for the extraction of DNA. Because there were less overall sample per individual available from the ITG, 30 mg was processed from that region while 100 mg of tissue was processed from the SFG. All handling of tissues was performed inside a biosafety cabinet to avoid potential contamination from the room air. To minimize potential microbial DNA contamination that may have occurred during post-mortem acquisition of the tissues at BSHRI, outer surfaces of the frozen tissue samples were carefully cut off with a sterile/DNA-free scalpel (Fisher Scientific, Hampton, NH, USA) prior to obtaining a subsample of the tissue for DNA extraction. To ensure the DNA-free nature of the scalpel and to prevent cross-contamination, a new sterile scalpel blade was used for each individual tissue. Before using the scalpels to trim outer surfaces, they were dipped in 100% ethanol (Thermo Fisher Scientific, Waltham, MA, USA) and flame-sterilized. During trimming of the tissues on dry ice, tissues were placed on sections of aluminum foil (Thermo Fisher Scientific, Waltham, MA, USA) that had been soaked in 10% Clorox household bleach (The Clorox Company, Oakland, CA, USA) for 1–2 h. Cut tissues were weighed in sterile DNAase/RNAase-free microfuge tubes (USA Scientific, Ocala, FL, USA).

DNA extraction was performed in a biosafety cabinet using the MoBio Powersoil DNA Isolation Kit (Cat. 12855-100), now available from Qiagen (Germantown, MD, USA), which used both chemical and mechanical (bead beating) lysis and captured DNA on a silica membrane. This method has been found to be highly effective for recovering microbial DNA from low-microbial-biomass samples [43]. The manufacturer’s protocol was followed, with one modification to adapt it for the high fat content of the brain tissue. The initial vortexing and homogenization step was followed by addition of phenol:chloroform (Thermo Fisher Scientific, Waltham, MA, USA), as suggested by the MoBio technical service. The remaining protocol steps were followed as written. Extraction of DNA from matched serum samples was also attempted but ultimately failed to provide sufficient DNA for analysis following removal of genomic DNA (see Microbial DNA Enrichment below).

### 2.3. Microbial DNA Enrichment for DNA Sequencing

Due to the high human genomic DNA content of the resulting eluent from the MoBio Powersoil DNA Isolation Kit, the extracted DNA was then enriched for microbial DNA using the NEBNext Microbiome DNA Enrichment Kit (New England Biolabs, Ipswich, MA, USA; Cat. E2612L). Samples were handled within a biosafety cabinet under sterile conditions. This kit removes CpG-methylated host genomic DNA, which makes up the majority of the extracted DNA from brain tissue. The removal of a large portion of host genomic DNA enables more effective sequencing of the largely un-methylated microbial sequences remaining in the extracted DNA. Because host mitochondrial DNA is predominantly methylated at non-CpG sites, it is not as effectively removed via this kit. Mitochondrial sequences remain and must be identified and removed from the analysis post-sequencing. For steps in the extraction protocol requiring water, microbial DNA-free water was used to avoid contamination with exogenous DNA sequences (Qiagen, Germantown, MD, USA; Cat. 338132).

### 2.4. Reagent Control Samples

When analyzing low-microbial-biomass tissues such as brain tissue, contaminating DNA sequences introduced at any step of the analysis can be problematic and interfere with detection of true signal. To assist in detecting these contaminating sequences, water blank reagent control samples were run through every step of the process from DNA extraction to sequencing in conjunction with the subject tissue samples being extracted and sequenced. The water blank source was the above-mentioned microbial DNA-free water from Qiagen, which, in conjunction with other careful tissue handling techniques, we have found to substantially reduce the presence of detectable contaminating sequences.

### 2.5. 16S rRNA Gene Sequencing and Initial Data Processing

16S rRNA gene sequencing and basic data analysis were completed at the TGen North sequencing facility in Flagstaff, AZ, USA, which houses TGen’s Pathogen and Microbiome Division. The concentrations of samples provided to TGen were in a narrow range (10–40 ng/µL), averaging 23 ng/µL. Bacterial DNA was quantitated by BactQuant assay [44]. As expected for low-microbial-biomass samples [43], bacterial DNA abundance was low, so the standard normalization to 200,000 bacterial copies/µL could not be performed and sample concentrations were not adjusted. 16S rRNA gene amplicon libraries were created by amplifying the variable region 4 (V4) using dual-index primers that included the Illumina adapters [45]. Amplicons were normalized, pooled, and sequenced on an Illumina MiSeq (2 × 250 bp) run (Illumina, San Diego, CA, USA). Mock community analysis and no template controls were included as internal quality control procedures. Primary sequence analysis was performed by TGen using QIIME2 (https://qiime2.org/, accessed on 1 May 2019) [46]. Operational taxonomic units (OTUs) were assigned using the SILVA taxonomy database. As the primers that were used also amplified host mitochondrial DNA, human mitochondrial sequences were removed during post-sequencing data analysis.

### 2.6. qPCR Analysis

qPCR for detection of viral DNA from HSV-1 and HHV6A and HHV6B was performed on subsamples of the DNA extracted from our subject groups for 16S rRNA gene sequencing. The HSV-1 qPCR used the nested outer and inner primers as well as the qPCR protocol described in Victoria et al., 2005 [47]. HSV-1 control DNA for qPCR standards was acquired from the American Type Culture Collection (ATCC, Manassas, VA, USA; #VR-1493D). In our hands, the lower limit of detection using this protocol was approximately 10 copies of the HSV-1 viral genome. A commercially available kit with included standards (Virusys Corporation, Milford, MA, USA; #H6K243) was used for the detection of HHV6A and HHV6B viral DNA as per the manufacturer’s protocol. qPCR was run using SYBR Green and ROX Passive Reference Dye (Thermo Fisher Scientific, Waltham, MA, USA).

### 2.7. Sample Preparation for ELISA Analysis

Subsamples of frozen SFG (400 mg), ITG (60 mg), liver (150 mg), and spleen (150 mg) samples were processed to create extracts suitable for ELISA testing. Briefly, samples were hand-homogenized in equal ratios of ice-cold sterile phosphate buffered saline (PBS) (Thermo Fisher Scientific, Waltham, MA, USA) containing a protease/phosphatase inhibitor cocktail (Thermo Fisher Scientific, Waltham, MA, USA). Homogenized samples were then sonicated on ice using a probe sonicator (Fisherbrand, Thermo Fisher Scientific, Waltham, MA, USA) in a biosafety cabinet at a 40% amplitude for a total time of one minute with alternating on/off sequences of 15 s. Samples were then centrifuged for 30 min at 14,000× *g* at 4 °C. The supernatant was stored at −80 °C until ELISA analysis. Serum samples used for ELISAs were thawed and diluted in ice cold sterile PBS (Thermo Fisher Scientific, Waltham, MA, USA) as necessary based on initial testing to align the concentration of analytes with the working range of the ELISA tests. For SFG, ITG, liver, and spleen samples, tissue was available from all subjects. For serum, *n* = 8 for normal controls, *n* = 11 for HPCs, and *n* = 12 for MCI and AD patients.

### 2.8. ELISA Analysis

ELISA kits (LifeSpan Biosciences, Shirley, MA, USA; #LS-F17912) were used to measure LPS and kits from Antibodies-online.com (Limerick, PA, USA, #ABIN2536149) were used to measure lipoteichoic acid (LTA) in brain tissue extracts. The same Lifespan Biosciences kits were also used to measure LPS in serum samples. Insufficient serum was available to perform LTA ELISA testing on subject serum samples. For LPS ELISAs using tissue extracts, samples were diluted either 1:500 or 1:4000 as necessary to bring them into the working range of the ELISA test. For LTA ELISAs using SFG or ITG extracts, samples were diluted 1:5 for analysis to bring them into the working range of the ELISA test. ELISA kits were also used for measuring levels of lipopolysaccharide binding protein (LBP; LifeSpan Biosciences, Shirley, MA, USA; #LS-F4664) from liver, C-reactive protein (CRP; Antibodies-online.com, Limerick, PA, USA; #ABIN6574220) from liver, and tumor necrosis factor-alpha (TNF-α; (LifeSpan Biosciences, Shirley, MA, USA; #LS-F24689) from spleen. Samples for these ELISAs were diluted as necessary to bring them into the indicated working range. All samples were run in duplicate or triplicate. A standard curve was run on each ELISA plate. OD values minus sample diluent blanks were analyzed using a 4-parameter logistic curve in GraphPad Prism (La Jolla, CA, USA).

### 2.9. 16S rRNA Gene Data Analysis

16S rRNA gene sequence data are, by nature, compositional and, therefore, not statistically appropriate for traditional alpha and beta diversity analyses [48,49,50,51,52]. We accounted for this compositional structure by employing ratio transformations to the non-rarefied, filtered sequence data, following the methods of Gloor [53]. We conducted all statistical analyses in R 4.1.2 (R 2022). All R code and associated read data have been published via figshare (doi:10.6084/m9.figshare.25902031). We filtered all reads that did not meet the minimum requirements of minimum reads = 0, proportional abundance minimum = 0.001 and proportional abundance maximum = 2, and minimum occurrence = 0.2 using CoDaSeq [54]. The resulting filtered data included the five phyla represented in all samples: Actinobacteria, Bacteroidetes, Cyanobacteria, Firmicutes, and Proteobacteria. All raw sequencing data files (fastq) have been uploaded to the NCBI Sequence Read Archive (SRA) and assigned BioProject accession number PRJNA1129202 (accessed on 28 June 2024). Following publication, these files can be accessed via the following link: https://www.ncbi.nlm.nih.gov/sra/PRJNA1129202.

#### 2.9.1. Relative Abundance

Explicit open-source bioinformatics software (http://www.explicet.org, accessed on 1 September 2019), version 2.10.5, was used to perform analysis on non-rarefied sequencing data comparing the mean relative abundance of the top 5 phyla across subject groups and with respect to brain regions (SFG versus ITG) using pie charts as graphical representations. Additional analysis compared the mean relative abundance of classes within the top 5 phyla, again with respect to subject group and brain region, using bar graphs as the graphical representation.

#### 2.9.2. Alpha Diversity

Alpha diversity is a means of quantifying the diversity of microbes within a group of samples. The alpha diversity within each group can then be compared to the alpha diversity in other groups of samples. We calculated the alpha diversity of each sample group as quantified by the Shannon index (richness) and Simpson index (evenness) [55] using vegan [56] and car [57]. We sought to determine whether the degree of diversity in bacterial phyla in subjects with significant brain pathology (HPC, AD) was different from the diversity in subjects without brain pathology (NDN). We also evaluated the degree of diversity in individuals that demonstrated cognitive decline (AD, MCI) and compared it to the degree of diversity in those that did not (HPC, NDN). We used a two-sample t-test to test for a significant difference in both Shannon and Simpson indices between pathology and non-pathology groups. We also used a two-sample t-test to determine whether there was a significant difference in the Shannon and Simpson indices between individuals with and without cognitive decline.

#### 2.9.3. Beta Diversity

Beta diversity is a means of evaluating whether the diversity of microbes differs between our sample groups. Prior to calculating beta diversity, we corrected the data using a centered log-ratio (clr) transformation [58]. This transformation was conducted in one of two ways, depending on the subsequent analysis. First, we calculated clr values via codaSeq.clr within the CoDaSeq package [54]. We also calculated clr values through the aldex.clr function in ALDEx2 [59,60]. This function generates Monte Carlo samples of the Dirichlet distribution for each sample and then converts them using the clr transformation.

#### 2.9.4. Principal Components Analysis

To visualize the distribution of variation in bacterial phyla within our sample set, we ran a standard principal components analysis (PCA) on the codaSeq.clr values. Due to the prominent nature of Proteobacteria across all our samples, we also sought to investigate variation across samples within orders of Proteobacteria. We therefore ran an additional PCA using codaSeq.clr values generated from orders within Proteobacteria alone.

#### 2.9.5. ANOSIM

We sought to determine whether the bacterial composition of our samples differed by group (AD, MCI, NDN, HPC), the brain region from which tissue was sampled (ITG, SFG), the presence or absence of pathology, or cognitive decline. We tested this using an analysis of similarities (ANOSIM) on the aldex.clr-generated clr values using the package vegan [56]. We ran each analysis for 999 permutations. When testing for differences between groups, we ran a series of pairwise ANOSIM, which is necessary due to the nature of the aldex.clr object. These analyses then included the following comparisons: AD vs. MCI; AD vs. NDN; AD vs. HPC; MCI vs. NDN; MCI vs. HPC; and NDN vs. HPC.

We tested for differences in bacterial composition among our samples strictly within Proteobacteria by using an ANOSIM on the aldex.clr-generated clr values for Proteobacteria orders using the package vegan [56]. Again, the same comparisons were made as with the phyla: group (AD, MCI, HPC, NDN), the brain region from which tissue was sampled (ITG, SFG), presence or absence of pathology, or cognitive decline.

#### 2.9.6. PERMANOVA

We also tested for differences in bacterial composition between groups (AD, MCI, HPC, NDN), the brain region from which tissue was sampled (ITG, SFG), presence or absence of pathology, and cognitive decline using a permutational multivariate analysis of variance (PERMANOVA) within vegan [56]. We calculated an Aitchison distance matrix of the codaSeq.clr-generated clr values using vegdist [56]. Again, we ran each analysis for 999 permutations. We tested for differences between groups through a multilevel pairwise comparison via pairwise.adonis [61].

We then tested for differences in the proportions of single phyla among groups and region to determine whether some phyla are more or less represented within a single group and/or region over others. To do this, we ran a PERMANOVA on the Aitchison distance matrix of the codaSeq.clr-generated clr values of each phylum individually with both group and region as factors. Again this analysis was run using vegdist [56] for 999 permutations.

We also tested for differences in bacterial composition among our samples strictly within orders of Proteobacteria. We tested this using PERMANOVA on the Aitchison distance matrix for 999 permutations using the package vegan [56]. Again, the same comparisons were made as with the phyla: group (AD, MCI, HPC, NDN), the brain region (ITG, SFG) from which tissue was sampled, pathology, or cognitive decline.

#### 2.9.7. Correlations between Phyla Based on 16S rRNA Sequencing

We tested for a correlation between bacterial phyla across our entire dataset as calculated through the correlation coefficient, rho (ρ) [62]. Specifically, we calculated the expected ρ (E(ρ)) using the aldex2propr function in propr [63]. E(ρ) as calculated through propr is robust to both sparse data and data subsetting [50,53,63], making it a more appropriate statistic for microbiome sequence data than traditional correlation metrics.

### 2.10. Correlations between Subject Characteristics, Pathology, and Peripheral Tissues

Spearman’s rank correlation coefficient was used to evaluate the strength of associations between subject characteristics, subject neuropathological features, and ELISA measurements of bacterial LPS and LTA, as well as for human LBP, CRP, and TNF-α. Analysis was performed using GraphPad Prism (9.5.1) software.

## 3. Results and Discussion

### 3.1. Subject Groups and Characteristics

Because there is a vast difference between conditions in the brains of AD patients versus controls, we opted to include additional subject groups in an attempt to more clearly define microbial influences associated with varying outcomes. As we were interested in whether certain types of microbes might initially provoke the pathology of AD, or alternatively, be protective against it, we included, in our study, individuals with MCI, which is often a precursor to the development of AD. Further, we were aware that some individuals have substantial AD-associated pathology in their brains but lack concomitant cognitive decline. We designated these individuals as HPCs. We hypothesized that this group may reveal whether certain bacterial taxa are beneficial or neutral, as opposed to being associated with processes resulting in cognitive decline. Tissue from a total of 48 subjects (*n* = 12/group) was used in this study as shown in Table 1. The average age of NDN subjects was not significantly different from the average age of AD patients (*p* = 0.7147). However, MCI and HPC subjects were older on average compared with both AD subjects (AD vs. MCI, *p* = 0.0601; AD vs. HPC, *p* = 0.0043) as well as NDN subjects (NDN vs. MCI, 0.0638; NDN vs. HPC, 0.0114). The ratio of male to female subjects in each group is shown here, as are the mean post-mortem intervals, which averaged approximately 3 h for all groups. The final recorded MMSE scores for these subjects are included in this table demonstrating a decline in score associated with cognitive/memory impairment in the MCI and AD groups. Also shown is the APOE status for the subjects in each group. The number of subjects with each indicated APOE allele genotype is denoted by the numbers shown. The post-mortem neuropathology of the subjects, as reported by the BSHRI, is shown, including plaque scores based on CERAD scoring, neurofibrillary tangle scores, and Braak staging. Plaque and tangle scores represent the numbers of subjects who scored none, sparse, moderate, or frequent. Brain tissue from these same subjects was also used in previously published metabolic profiling experiments [64], which included, as a primary author, one of the authors of this study (GJ).

### 3.2. Minimal Detection of HSV-1, HHV6A, and HHV6B Viral DNA in the Superior Frontal and Inferior Temporal Gyri

qPCR analysis was run on DNA extracted from our samples to detect the presence of HSV-1, HHV6A, and HHV6B. The presence of these viruses has been previously linked to AD in various reports [5,27,29,31,32]. In the present study, very few samples had detectable HSV-1 viral DNA. Specifically, in the analysis of DNA extracted from SFG tissues, there were no positive samples while, in the analysis of DNA extracted from ITG tissues, there were three positive samples, including one individual each in the MCI, HPC, and NDN groups, with the individual in the MCI group showing the strongest signal. No individuals in the ITG AD group were positive for HSV-1 viral DNA. For HHV6A and HHV6B analysis in both the SFG and ITG samples, no viral DNA was detected in any group, consistent with what others have observed [65]. From this analysis, we conclude that while some HSV-1 viral DNA was detected in a small number of our tissue samples, it was distributed over multiple groups and no association with a specific subject group can be made. Further, we detected no HHV6A or HHV6B viral DNA in any of the 48 SFG and 48 ITG samples, leading us to conclude that these viruses are unlikely to influence AD-associated pathology.

### 3.3. 16S rRNA Gene Sequencing Identifies Brain Region as an Important Variable in Determining Microbial Presence

#### 3.3.1. Bacterial Read Counts

**Figure 1:**
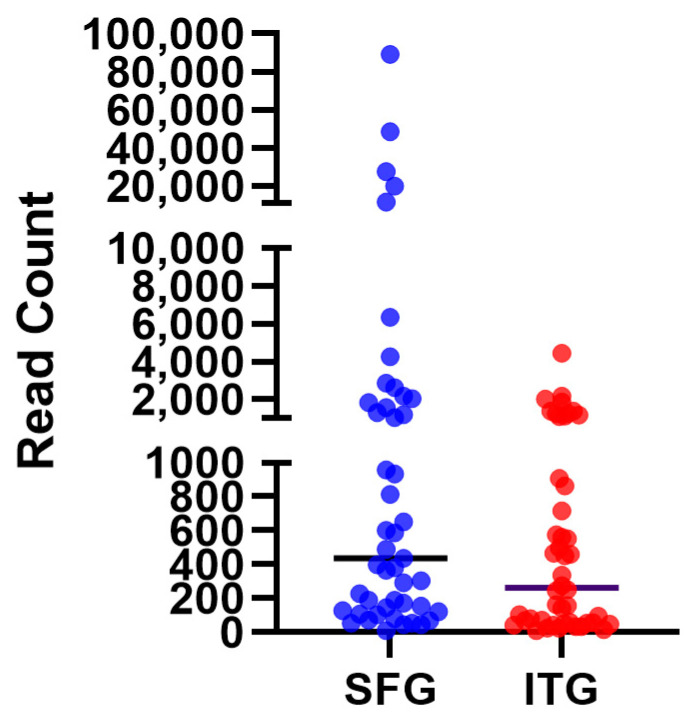
Bacterial 16S rRNA gene sequencing read counts for DNA extracted from superior frontal gyrus (SFG) and inferior temporal gyrus (ITG) tissues after removal of human mitochondrial DNA sequences. Black line indicates median values. Vertical axis scales are 0–1000, 2000–10,000, and 20,000–100,000 reads. Significant outliers were noted in data from SFG tissue.

As expected for a low-microbial-biomass tissue such as the brain, overall read counts were low in comparison to those from other types of microbiome analyses, such as those assessing gut or soil microbiomes. We were able to identify bacterial sequences from all 48 SFG samples and from 46 of the 48 ITG samples. Prior to the removal of human mitochondrial sequence reads, the SFG tissue samples cumulatively contained approximately double the number of reads (464,609) compared to the ITG tissue samples (254,181). Differing amounts of tissue from the two regions were used for extraction and intrinsic levels of mitochondria between these two regions could potentially vary. After the removal of human mitochondrial sequences, SFG tissue averaged 4969 reads per sample and ITG tissue averaged 600 reads per sample although the median values were similar between the two regions (Figure 1). The analysis of reagent control samples revealed that they contained reads from only three bacterial species, which were present at trace levels: 13, 7, and 6 total reads. Two of these species were almost entirely absent from experimental samples. However, the species with seven reads (found in a control run specifically with the ITG samples) was present in nearly all ITG samples and a portion of the SFG samples, albeit at an average read count of ~150, which might suggest that a small number of sequences from the experimental samples contaminated the associated control rather than the control detecting true reagent contaminants.

#### 3.3.2. Evaluation of Bacterial Diversity Based on Subject Group, Brain Region, Pathology, and Cognitive Status

Because a loss of microbial diversity has been reported for several neurological and other chronic inflammatory conditions including Alzheimer’s and Parkinson’s diseases, traumatic brain injury (TBI), and chronic rhinosinusitis [33,39,66,67], we evaluated the extent of bacterial diversity using both alpha and beta diversity measures. Alpha diversity assessed how diverse the bacterial phyla were within our subject groups whereas beta diversity was used to quantify the degree of diversity in bacteria that existed between our subject groups.

##### Alpha Diversity Measures Do Not Correlate with Neuropathology or Cognitive Status

We found alpha diversity, as quantified through the Shannon index to indicate a reasonable richness of taxa given the inherent low-biomass nature of brain tissue, with average values across all samples of 0.67. We found no significant differences in the Shannon index between individuals with and without pathology (*p* = 0.2256), nor between those with and without cognitive decline (*p* = 0.5751). Simpson index values also indicated that our samples were relatively diverse with an average of 0.38 across individuals. Using Simpson index values, there was again no significant difference between individuals with and without neuropathology (AD/HPC vs. NDN, *p* = 0.464) or with and without cognitive decline (AD/MCI vs. HPC/NDN, *p* = 0.5082). Note that MCI patients do typically have AD-associated pathology, but it was more variable than in our AD and HPC groups and was therefore excluded from the analysis of neuropathology versus alpha diversity. We conclude that neither the common AD-associated neuropathology nor declining cognitive status is influenced by the diversity of bacterial phyla within these subject groups.

##### Beta Diversity of Microbial Infiltrates Is Influenced by Brain Region and Pathology

**Figure 2:**
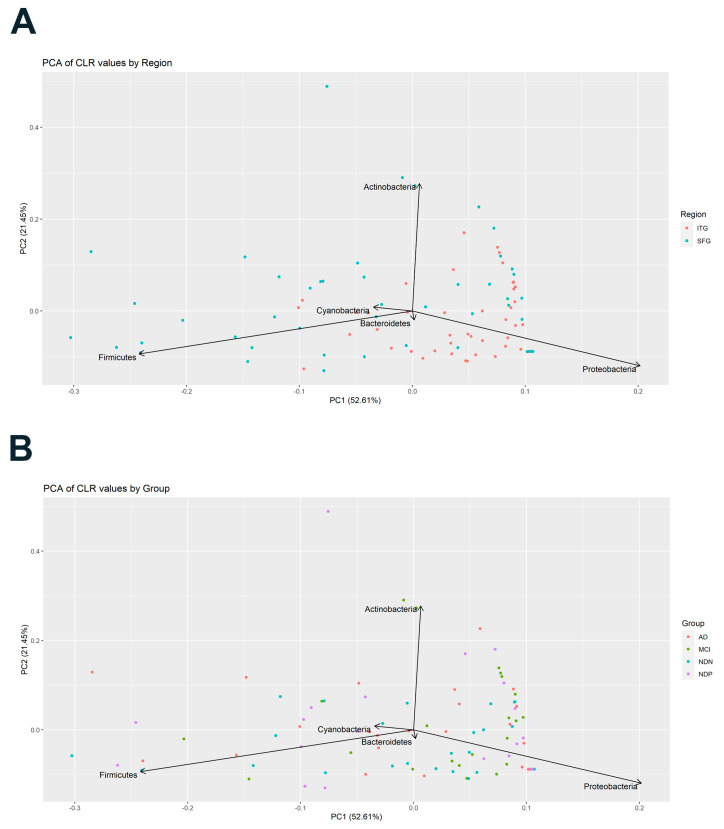
Principal components analysis (PCA) of the top five phyla present in the brain tissue of subjects. In (**A**), data are separated by tissue region, ITG versus SFG. In (**B**), data are separated by subject group and include both tissue regions.

We used PCA to assess beta diversity in microbial infiltrates across all samples. We found that PC1 explained 52.61% of the variance in bacterial phyla across samples, with the greatest variation being between the proportion of Proteobacteria and Firmicutes. This specific variance appears to distinguish ITG and SFG samples, with the ITG having higher proportions of Proteobacteria and the SFG having a more predominant Firmicutes signal (Figure 2A). PC2 explained 21.45% of the variation across samples, with Actinobacteria being a major driver on that axis, but did not clearly distinguish region (Figure 2A) or subject group (Figure 2B). As seen in the PCA plot in Figure 2B, individual subjects in all of our groups demonstrated a considerable degree of variability in bacterial relative abundance across the five most prominent bacterial phyla, which was not unexpected given the results of the alpha diversity analyses.

**Figure 3:**
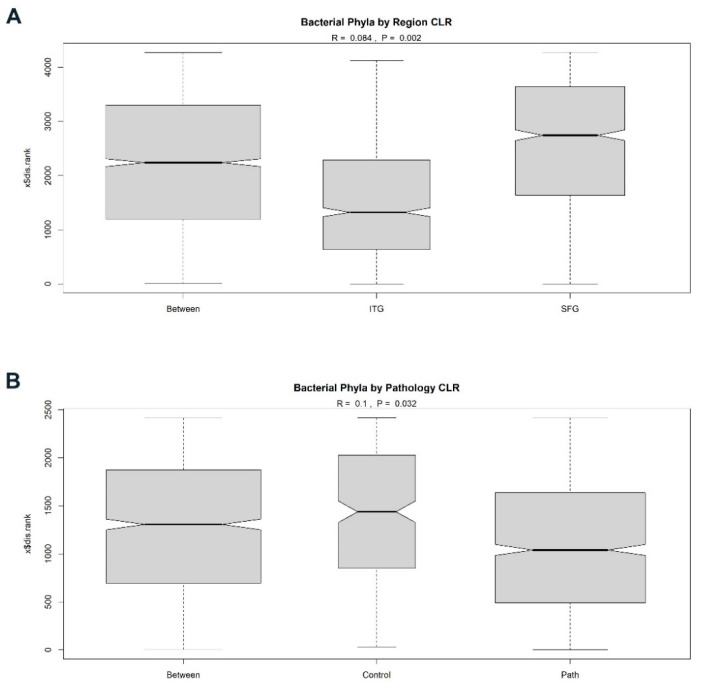
Analysis of similarities (ANOSIM) of the five major bacterial phyla based on region (**A**) and by the presence or absence of AD-associated neuropathology (**B**).

Using ANOSIM, we identified a significant but modest separation between the bacterial phyla found in ITG versus SFG samples (Figure 3A: r = 0.0835, *p* = 0.002; Table 2), with the SFG having a higher within-group dissimilarity than the ITG, suggesting greater diversity of bacterial phyla in that region. We also noted a modest but significant separation in bacterial phyla between individuals with AD-associated neuropathology (AD/HPC) and those without it (NDN) (Figure 3B: r= 0.0979, *p* = 0.032; Table 2). Specifically, individuals with neuropathology had lower within-group dissimilarity than control individuals lacking substantial neuropathology. This suggests a decrease in bacterial diversity in association with pathology. We did not, however, find a similar difference between individuals with and without cognitive decline (Table 2), indicating that cognitive status and pathology may not be closely linked in terms of the influence of varying bacterial phyla. In considering neuropathology, it is possible that the presence of plaques and tangles alters the environment in a manner that affects the microbes that accumulate in the tissue, or, conversely, that the microbes could affect the local environment in a manner that influences the accumulation of these pathological hallmarks, which would be in keeping with an antimicrobial role for pathological molecules. We also performed an ANOSIM of tested group pairings (AD vs. MCI, AD vs. HPC, AD vs. NDN, MCI vs. HPC, MCI vs. NDN, and NDN vs. HPC) but did not identify significant separation between those paired groups.

Analysis of the data by a second statistical technique, PERMANOVA, also indicated that there is a strongly significant difference in bacterial phyla between the regions (*p* = 0.0002; Table 2), supporting the ANOSIM. However, by PERMANOVA, we found no significant differences in bacterial phyla between individuals with and without neuropathology, in contrast to the ANOSIM, highlighting differences between these two statistical tests. In agreement with the ANOSIM, there was no significant difference between individuals with and without cognitive decline (Table 2). Similarly, there were no significant differences in phyla between individual subject groups for any of these measurements.

Collectively, these data indicate that the SFG and the ITG are significantly different from each other with respect to the composition of the five major phyla that are found in these regions of the brain. In addition, there is a significant decrease in bacterial diversity in the ITG compared to the SFG and in individuals with neuropathology compared to those without. Other investigators have found that the presence of neuropathology is associated with a reduction in the diversity of microbial infiltrates in the brain in AD [33] and other neurological conditions, including Parkinson’s disease and TBI [39,66]. While it is not possible to specifically determine the impact of reduced microbial diversity in individuals with higher levels of pathology, a decrease in microbial diversity has been implicated in the induction of inflammation and infection [68], which are common features in AD. We did not see any change in diversity in relation to cognitive decline, perhaps suggesting that the microbes could be associated with the presence of specific pathologies but not with cognitive outcomes. Given that others have found that a wide variety of microbes can induce the amyloid pathology seen in AD [4,69,70], this is perhaps not surprising. Ultimately, we were unable to conclusively link any specific bacteria or bacterial taxa to the presence of either AD or MCI.

#### 3.3.3. The Relative Abundance of the Five Major Phyla Differs in the SFG and ITG

**Figure 4:**
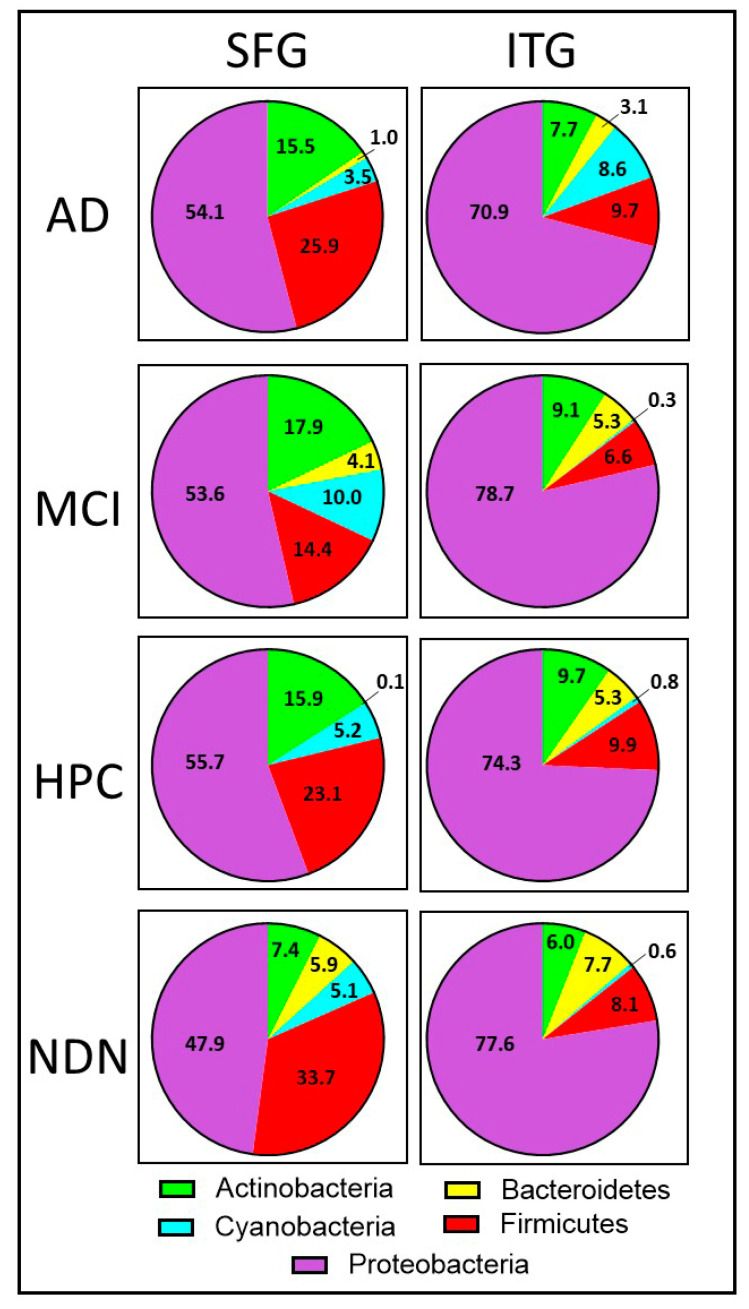
Comparison of the relative proportions of the five major phyla across subject groups between the SFG (**left panels**) and the ITG (**right panels**). Proteobacteria was the dominant phylum in all groups and was particularly abundant in ITG tissue.

Because the results of our ANOSIM and PERMANOVA indicated that the regions differed significantly in the top five phyla, we looked at the relative abundance of each of the phyla within the SFG and compared it to that within the ITG. We found that among the top five phyla represented across our entire sample set, both tissues were dominated by the presence of Proteobacteria; however, the proportion of Proteobacteria in all subject groups in the SFG was ~50% while in the ITG, it was ~75%. Comparison of the two regions (SFG and ITG) by PERMANOVA revealed that Firmicutes (*p* = 0.002) and Proteobacteria (*p* = 0.001) differed significantly in the SFG and ITG samples (Figure 4 left panels and right panels, respectively; Table 3), with Firmicutes representing a significantly greater proportion in the SFG compared with the ITG while Proteobacteria was more prevalent in the ITG (~75%) versus the SFG (~50%). We also compared the relative abundance of our top five phyla across our four subject groups (AD, MCI, HPC, and NDN) and found that there were no significant differences (see Table 3).

#### 3.3.4. Associations between the Five Major Phyla

Because of the differences in the distribution of five major microbial phyla between brain regions, we wanted to evaluate the strength of associations between the phyla themselves, which was important due to the propensity of bacteria to form mixed communities. Because our 16S rRNA data were compositional in nature, we utilized the rho (ρ) metric, which is more appropriate for this type of data, to evaluate associations between the five major phyla present in our samples [53]. We found moderate negative correlations between the majority of the phyla among the full sample set, with E(ρ) values ranging between −0.34 and −0.06 (Table 4), with the exception being a weak positive correlation between Actinobacteria and Proteobacteria (E(ρ) = 0.079; Table 4). The strongest negative correlations were between Actinobacteria and Bacteroidetes versus Cyanobacteria and Firmicutes (−0.31 to −0.34), suggesting that those two sets of phyla are less likely to be found in each other’s presence. Conversely, Proteobacteria was nearly neutral in its association with Actinobacteria and Bacteroidetes, but its presence was negatively correlated with the presence of Cyanobacteria and Firmicutes.

**Figure 5:**
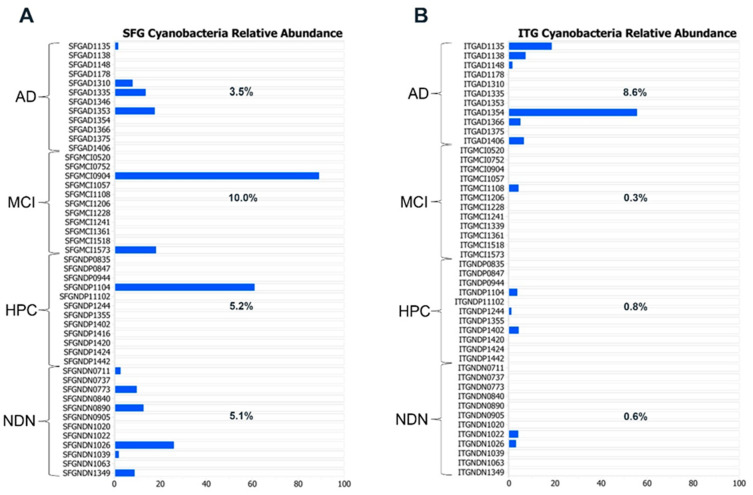
Percent relative abundance of Cyanobacteria in individual subjects in all subject groups in the SFG (**A**) and the ITG (**B**). Percentages shown on the graph indicate the average relative abundance of Cyanobacteria in each group.

As Cyanobacteria are typically regarded as widely distributed, primarily waterborne photosynthetic microbes, we were initially surprised to find them in our tissue samples. In our reagent control samples, the Cyanobacteria phylum was absent, making it unlikely that it was a simple water contaminant. Indeed, Cyanobacteria have been found to be normal inhabitants of both the gut [71,72] and respiratory tract [73]. We found that roughly 25% of brain tissue samples overall contained Cyanobacteria, and in three samples, it was the predominant phylum (Figure 5). In SFG tissue (A), Cyanobacteria was found in all subject groups, generally at a relative abundance of less than 30% when present. Cyanobacteria was also found in all groups when considering ITG tissue (B) but was present in the greatest frequency in AD tissues (six out of eleven samples). The frequency of Cyanobacteria-containing samples in the AD group could suggest an alteration in the environment in these individuals that allows for the persistence of Cyanobacteria. The presence of Cyanobacteria in all subject groups was interesting in view of previous reports implicating Cyanobacteria toxins in Alzheimer’s disease [74,75,76].

#### 3.3.5. Microbial Signatures at the Class and Order Levels Are Region-Specific but Are Not Influenced by Pathology or Cognitive Status

**Figure 6:**
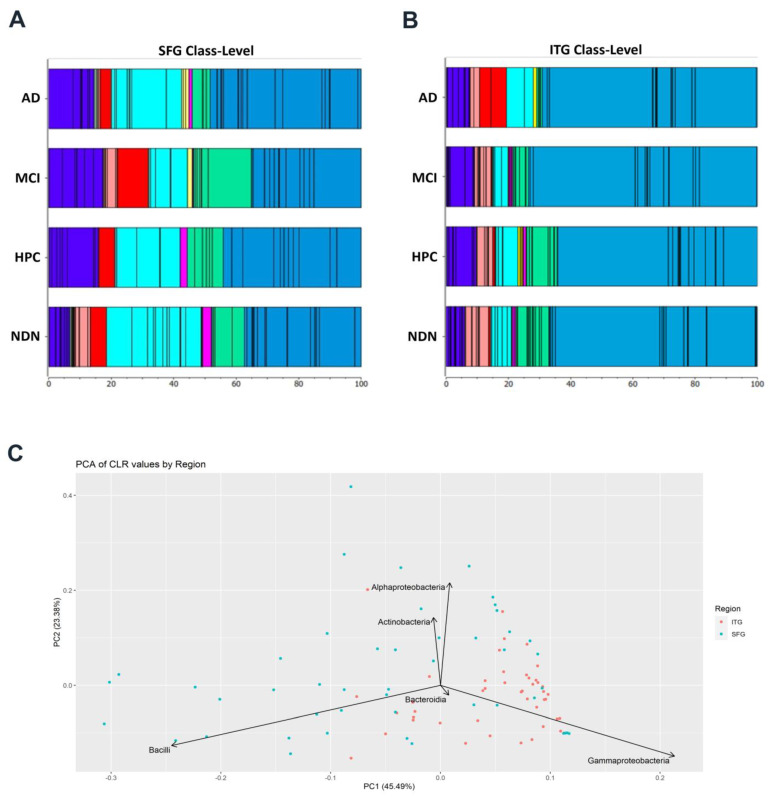
Distribution of bacterial classes between the SFG (**A**) and the ITG (**B**). PCA of bacterial classes between the two brain regions (**C**). In (**A**), dark blue bands on the far left represent multiple orders of the Actinobacteria class within the Actinobacteria phylum. The immediately adjacent narrow green/tan bands represent three additional orders of the Actinobacteria class, and pink bands show multiple orders of the Bacteroidia class of the phylum Bacteroidetes. Red bands were identified as the Oxyphotobacteria class of the Cyanobacteria phylum. Aqua, yellow, and fuchsia bands represent orders of the Bacilli, Clostridia, and Negativicutes classes of the Firmicutes phylum, respectively. The Proteobacteria phylum is shown in the light green bands, representing orders of the Alphaproteobacteria class, and also in blue (far right), representing orders of the Gammaproteobacteria class. In (**B**), color blocks represent the same classes and orders of bacteria as described for the SFG. Panel (**C**) is a PCA plot showing the classes of bacteria that explain the greatest variance between the SFG and ITG.

We evaluated the relative abundance of the five major bacterial phyla at the class level in the SFG (Figure 6A) versus the ITG (Figure 6B). In these figures, the major classes are represented by differing colors, and dividing lines within color blocks represent the relative abundance of orders within those classes. The collective Proteobacteria classes (green and blue color blocks furthest to the right) dominated both the SFG and ITG. However, the relative abundance of Gammaproteobacteria (blue color blocks) was much greater in the ITG and was in large part composed primarily of two particular orders of Gammaproteobacteria, Betaproteobacteriales and the Pseudomonadales. Betaproteobacteriales was also the dominant Gammaproteobacteria order in the SFG; however, the Pasteurellales order was more prominent in that tissue than Pseudomonodales. Pasteureallales had a much lower relative abundance in the ITG compared to the SFG. Collectively, this suggests that while Gammaproteobacterial classes are common in both tissues, there are notable differences in the abundances of specific orders within those classes. Classes belonging to the Actinobacteria, Bacteroidetes, Cyanobacteria, and Firmicutes phyla (found to the left of the green color blocks) made up a much greater relative proportion of the SFG samples. Within the Firmicutes phyla in SFG tissue, the Bacilli class was particularly prominent (aqua color blocks). Likewise, the Oxyphotobacteria class (red color blocks) of the Cyanobacteria phylum made up a larger share of the bacteria in SFG tissue compared to ITG tissue. Overall, the lower abundance of Proteobacterial classes in the SFG allowed other classes of bacteria to be more prevalent. Using PCA at the class level, we found that PC1 explained 45.5% of the variation between the SFG and ITG, which was driven largely by differences between the Gammaproteobacteria and Bacilli classes (Figure 6C). PC2 explained 23.4% of the variance with Alphaproteobacteria and Actinobacteria driving variance from Gammaproteobacteria and Bacilli.

**Figure 7:**
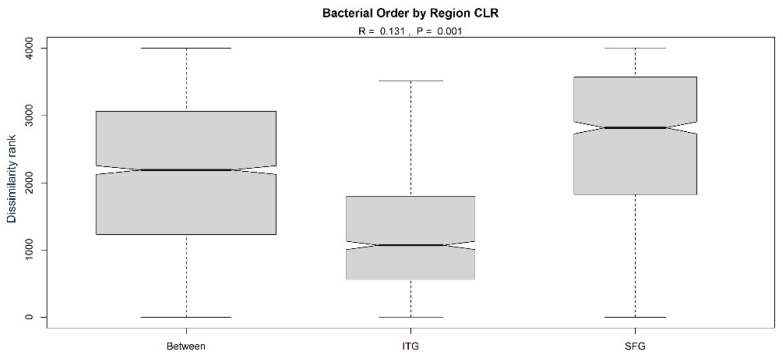
ANOSIM comparing differences between Proteobacterial orders found in ITG versus SFG tissue (R = 0.131, *p* = 0.001).

Based on the class-level analysis that identified Proteobacteria as a driving force of the regional variances, and given the predominance of Proteobacteria in both the SFG and ITG, we proceeded to evaluate regional differences specifically across Proteobacterial orders. We used ANOSIM to assess whether the differences were statistically significant (Figure 7). Consistent with our results described above (Figure 6A,B), the analysis identified a significant (*p* = 0.001) but moderate (r = 0.131) difference between Proteobacterial orders from the ITG versus the SFG samples. We subsequently extended our statistical analyses to differences in Proteobacterial class order composition between individuals with and without AD-associated brain pathology or between individuals with and without cognitive decline, irrespective of tissue region. In both cases, no significant differences were identified either by ANOSIM or by PERMANOVA (Table 5).

Overall, the distribution of bacterial DNA we identified in brain tissue from our subjects aligned well with the data previously published by others. Specifically, Proteobacteria, Actinobacteria, Firmicutes, and Bacteroidetes phyla have been consistently described as predominant in various brain regions in AD studies [33,36,37,40]. Cyanobacteria [33,36] and Fusobacteria [37] have also been identified as major phyla in the brain.

Collectively, in combination with our own data, there is substantial evidence that these findings of bacterial DNA in brain tissue are real and relatively reproducible at the phylum level from study to study. While most studies have used tissue from a single brain region, our data demonstrated that different brain regions from the same individuals have different microbial profiles, consistent with the findings of Hu et al. 2023 [41]. Our work extends these findings and shows that individual brain regions have relatively consistent microbial profiles, particularly at the phylum and class levels, across four subject groups with a range of pathology and cognitive impairment, suggesting that the microenvironment rather than pathology drives the microbial profile within brain regions.

The predominance of Proteobacteria in the brain is particularly interesting in that it is very consistent across studies of brain tissue and not the most dominant phylum in other commonly studied microbiome sites. When assessing various commonly studied microbiomes, including those in stool, on skin, and in oral and nasal cavities, Proteobacteria phylum bacteria are in higher abundance on the skin and in the oral cavity [77,78], making those sites potential sources of the bacteria found in the brain. While the gut is a major microbiome site, Hu et al. 2023 [41], found little overlap between the microbes present in the brain compared with those found in the gut, which are predominantly Bacteroidetes and Firmicutes. Interestingly, the blood microbiome is dominated by the Proteobacteria phylum, with a relative abundance of greater than 80% [79]. However, in the blood, Alphaproteobacteria is the predominant class observed [79], while in contrast, in the brain, we observed that the Gammaproteobacteria class was much more prevalent. As a particularly likely route of entry into the brain is via the blood–brain barrier, Gammaproteobacteria may be more successful in gaining entry than Alphaproteobacteria.

### 3.4. Direct Confirmation of the Presence of Non-DNA Bacterial Components in the Brain

**Figure 8:**
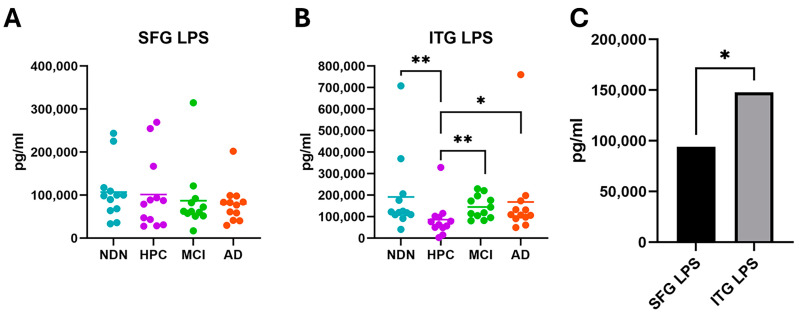
Identification of LPS in the SFG (**A**) and the ITG (**B**) by ELISA. Note the differing scales of the Y axis in these figures. Horizontal bars indicate mean values within each subject group. No significant differences between groups were identified in the SFG tissue. In the ITG, LPS levels in the HPC group were significantly lower than in all other groups. A comparison of mean overall levels of LPS levels in each tissue is shown in (**C**). Levels were significantly different between the two tissues. * *p* = 0.05, ** *p* = 0.01.

To validate our DNA sequencing findings, we used adjacent subsamples of the same tissues previously used for 16S analysis to perform ELISA analyses with the goal of detecting the presence of the microbial cell wall constituents, LPS (Gram-negative bacteria) and LTA (Gram-positive bacteria). We found these molecules in remarkably high levels (particularly LPS) in brain tissue regardless of cognitive status (AD and MCI versus HPC and NDN) (Figure 8).

Measurements of LPS were not significantly different between groups in the SFG tissue (Figure 8A). However, in the ITG, LPS levels were significantly lower in HPCs than in all other groups (Figure 8B). As HPC and AD patient samples both had high levels of pathology, this difference in LPS content suggests that pathology and tissue LPS levels are not directly associated. Comparatively, LPS levels in the ITG were significantly higher overall than in the SFG (Figure 8C), in agreement with the greater predominance of Gram-negative bacteria in the ITG—Proteobacteria in particular, but also Bacteroidetes and Cyanobacteria (see Figure 4). Collectively, these findings are consistent with and confirm our sequencing data showing that microbial infiltrates are more closely associated with region than pathology or cognitive status, as indicated in Figure 6 and Figure 7 and Table 5.

**Figure 9:**
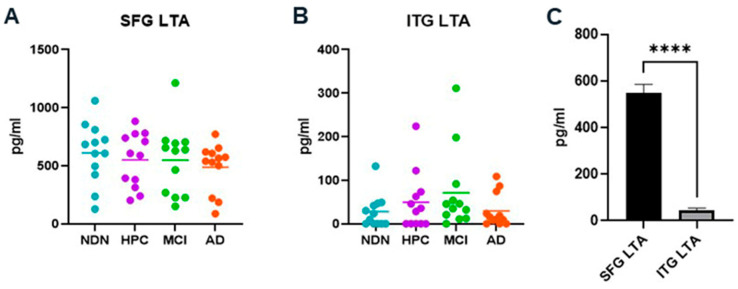
Identification of LTA in the SFG (**A**) and the ITG (**B**) by ELISA. Note the differing scales of the Y axis in these figures. Quantification of bacterial LTA (**C**). Horizontal bars indicate mean values within each subject group. No significant differences between groups were identified in either SFG or ITG tissue. However, the mean overall levels of LTA were significantly and notably different, with SFG having levels roughly 10 times higher. **** *p* = 0.0001.

LTA levels in the SFG (Figure 9A) versus the ITG (Figure 9B) corresponded well with the overall relative abundance of Gram-positive (Firmicutes (except Negativicutes class) and Actinobacteria) versus Gram-negative bacterial phyla (Proteobacteria, Bacteroidetes, and Cyanobacteria) detected by 16S rRNA analysis in the tissues (see Figure 4). Overall levels of LTA in the SFG were dramatically higher and statistically different from the LTA levels in the ITG (Figure 9C). It is notable that in terms of pg/mL, LTA levels, regardless of tissue, were very low compared with the overall levels of LPS in those same tissues (see the y-axis scales of LPS (Figure 8) and LTA (Figure 9) graphs).

#### Comparison of Relative Abundance of Gram-Negative and Gram-Positive Bacterial DNA via 16S rRNA Sequencing to LPS and LTA via ELISA

**Figure 10:**
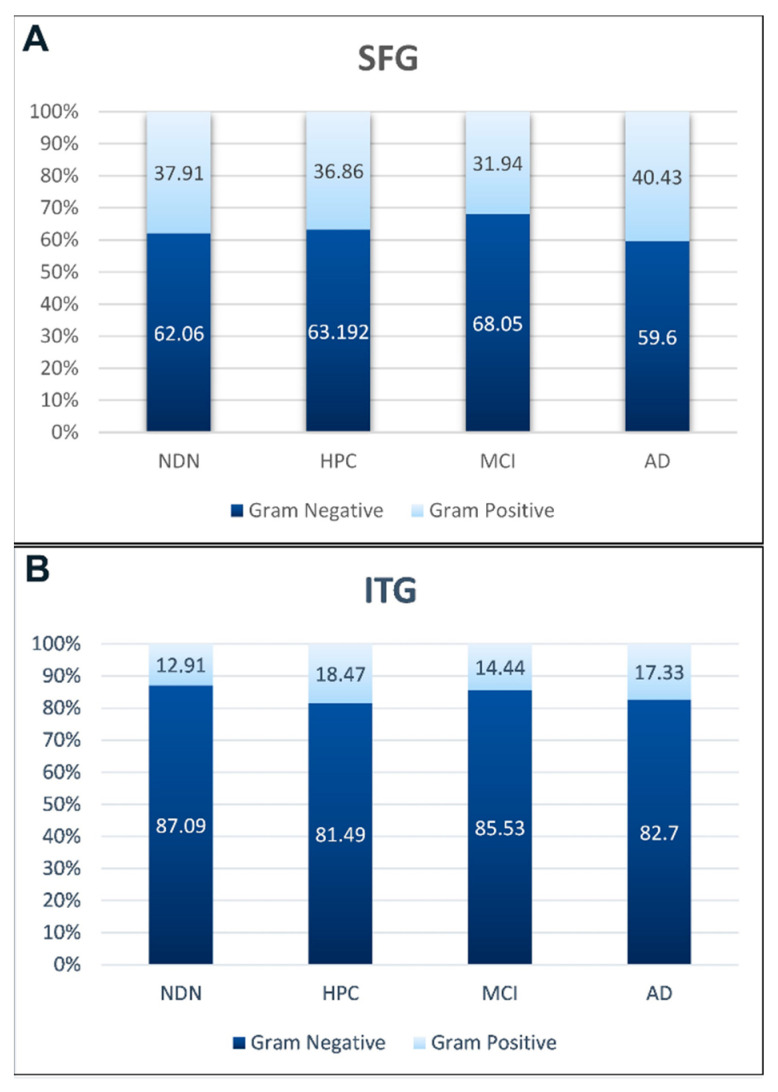
Comparison of the relative abundance of Gram-positive versus Gram-negative bacteria in SFG (**A**) and ITG (**B**) brain tissue. Light blue bars represent the proportion of Gram-positive bacteria as determined using 16S rRNA gene sequencing relative abundance data. Dark blue bars represent the proportion of Gram-negative bacteria. Gram-positive bacteria include bacteria of the Actinobacteria and Firmicutes phyla, except for the Gram-negative Negativicutes class of Firmicutes. Gram-negative bacterial bars include the Negativicutes class in addition to bacteria of the Bacteroidetes, Cyanobacteria, and Proteobacteria phyla.

Given that our tissue levels of LPS and LTA appeared to closely replicate what we described above for sequencing, we separated our five major phyla into either Gram-positive or Gram-negative bacteria. Figure 10A,B show the average relative abundance of Gram-negative (dark blue) versus Gram-positive (light blue) bacterial DNA in the SFG and ITG brain tissue samples as determined by 16 rRNA gene sequencing. As is shown in the figures, the proportion of Gram-negative to Gram-positive bacteria was much higher in the ITG, consistent with the LPS and LTA ELISA data shown in Figure 8 and Figure 9. This close agreement between the data provided by rRNA gene sequencing and the data provided by the ELISA analysis of LPS and LTA provides independent validation of the presence of bacteria in the brain and the variation of bacteria between brain regions.

### 3.5. Serum LPS, but Not Tissue Acute Phase Proteins, Is Elevated in AD and HPC Subjects

**Figure 11:**
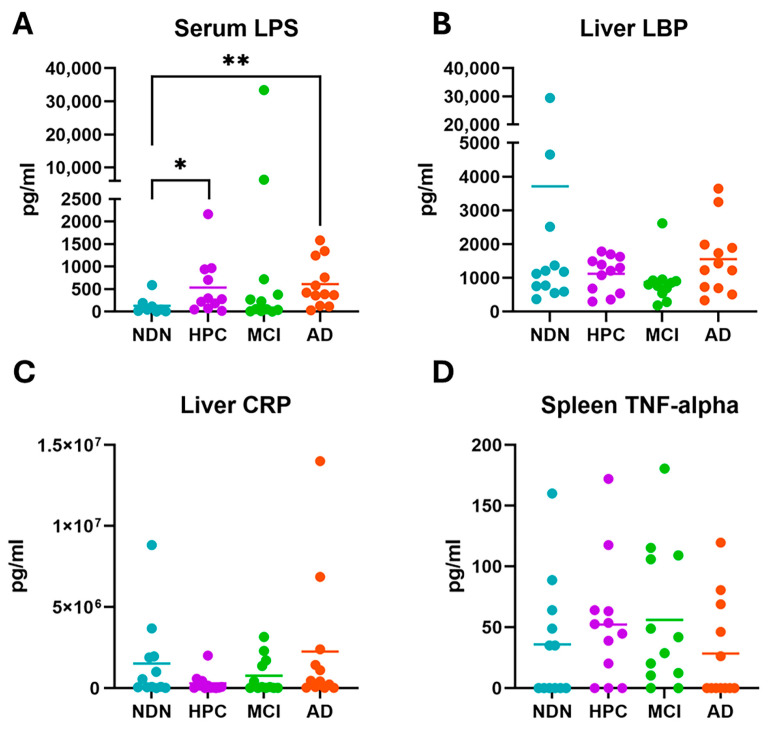
Evaluation of serum LPS (**A**) and acute-phase proteins in the liver (**B**,**C**) and spleen (**D**). Bars indicate mean values within each subject group. The mean value for serum LPS (A) in the MCI group = 3461 pg/mL but this is not shown due to axis break on the graph. The elevated mean in this subject group is influenced by the two high outliers previously mentioned. The median value for the MCI group was 140.6 pg/mL. *p* = 0.05 (*), *p* = 0.01 (**).

We sought to assess the relationship between findings in brain tissue samples in comparison with a variety of peripheral factors in our subjects as a means of assessing whether those aspects might be interrelated, perhaps for their potential use as biomarkers [80]. The peripheral factors chosen for evaluation are known to be associated with microbial presence or infection in the body, as well as with other drivers of inflammation. When comparing subject groups, serum LPS was found to be significantly higher in both HPC and AD subject samples than in NDN control samples (Figure 11A). During the analysis, we noted two particularly high LPS outliers in the MCI group (6345 pg/mL and 33,425 pg/mL). Thus, while the MCI group was also significantly elevated compared with the NDN control samples, this was due to the two outliers rather than an overall group difference.

Liver levels of LBP, which is produced by the liver, adipose tissue, and cells of the intestine [81] in response to Gram-negative bacterial infections, were not significantly different between any subject groups (Figure 11B). Liver CRP, which is produced in response to infection, chronic disease, or injury [82], was also not significantly different between subject groups (Figure 11C), in contrast to serum CRP, which has previously been shown to be decreased in AD [83,84]. Likewise, spleen levels of TNF-alpha, an inflammatory cytokine that is produced by macrophages in both the spleen and liver, were measured (Figure 11D), but again, we found no significant differences between subject groups. Note that while it would have been possible to measure LBP, CRP, and TNF-alpha levels in serum as well, we did not have sufficient post-mortem serum from the subjects to support those analyses, so tissues responsible for their production were analyzed instead.

**Figure 12:**
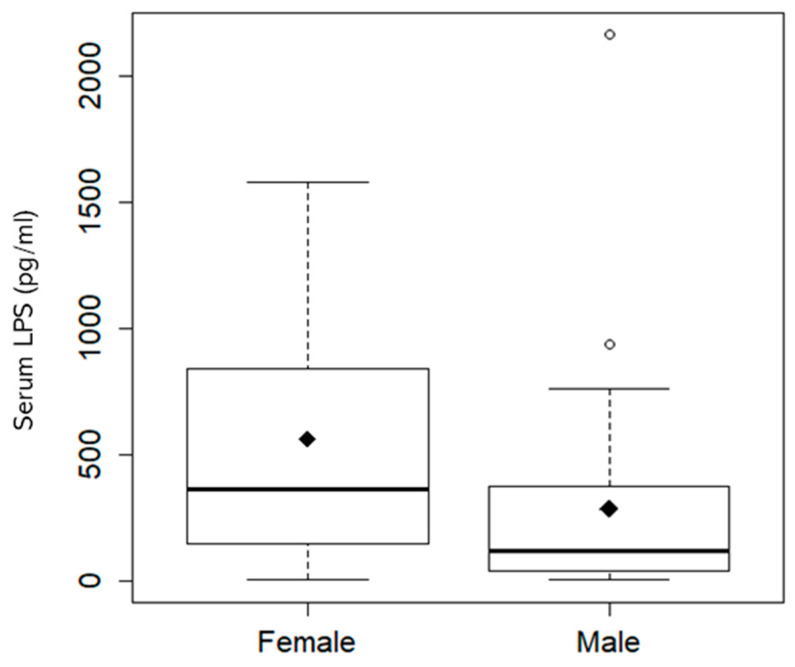
Serum LPS, shown in pg/mL, is significantly higher in females compared with males (*p* = 0.0143). Two high outliers (*p* < 0.05 by independent Grubb’s tests) in the MCI female group (6345 and 33,425 pg/mL) were removed from this analysis to prevent skewing of the results. Despite their removal, the difference between males and females remained significant. Open circles represent data points outside the upper quartile for males. The black line indicates median values and the solid black diamond represents mean values for each group.

Notably, we found that serum LPS showed a significant effect of sex; specifically, females had higher levels of LPS in serum compared with males across all samples. When running this statistical analysis, we observed that the two substantially higher values previously noted in the MCI group were both from female subject samples. Thus, we removed those two values as outliers and found that the difference between female and male subjects remained significant (Figure 12; *p* = 0.0143).

### 3.6. Correlation Analyses of Serum and Tissue LPS with Other Variables

#### 3.6.1. Serum LPS Does Not Predict Levels of LPS in Brain Tissue and Is Not Related to Subject Age

It has been suggested that LPS in brain tissue may be derived from free LPS in the bloodstream, which might to a large degree be derived from gut bacteria [85,86]. The presence of LPS in the bloodstream, even in healthy individuals, has been well documented. However, the question remains whether the LPS we measured in brain tissue was a result of bacterial presence in the brain (as the associated presence of bacterial DNA would suggest) or whether the LPS measured in the brain was a result of LPS molecules infiltrating the brain via the bloodstream and accumulating there.

After establishing the serum levels of LPS in our subjects, we checked for correlations between their serum LPS and the LPS measured in their two brain regions. Because we had previously noted two very high serum LPS outliers in the MCI subject group, we performed Spearman’s correlations using the collective set of LPS values from all subjects in the study both including and excluding those outlier values. Because a weakening blood–brain barrier associated with age could hypothetically allow more circulating LPS to deposit in the brain, we also looked at whether there was a correlation between serum LPS and age. There was no significant correlation found between the serum LPS in our subjects and the SFG and ITG brain tissue levels of LPS within those same subjects (Table 6). This suggests that circulating LPS levels, at least around the time of death, do not directly influence LPS levels in the post-mortem brain. This may be important as it has been suggested that peripheral LPS can enter the brain, complicating the assessment of whether brain LPS derives from microbes that are present in the brain versus microbes found elsewhere in the body, or possibly both [87]. LPS levels in the serum are also very low in most individuals compared to the high brain levels detected and would therefore need to accumulate over an extended period of time if the source was the bloodstream. Correlation analysis of serum LPS versus subject age indicated that subject age did not correlate with levels of LPS found in brain tissue, either with or without serum LPS outliers included (Table 6).

#### 3.6.2. Serum LPS Correlates with Lower MMSE Scores and Higher Amyloid and Tangle Pathology

We assessed whether significant correlations existed between the levels of serum LPS and pathological AD hallmarks, including plaques, tangles, CAA, or Braak score, and MMSE score (Table 7). Interestingly, MMSE scores negatively correlated with serum LPS levels both with (r = −0.374; *p* = 0.018) and without (r = −0.435, *p* = 0.006) the high LPS outliers included. This indicates that lower MMSE scores, which are indicative of cognitive decline, correlate with raised LPS levels in the serum. Serum LPS levels did not significantly correlate with Braak scores when high LPS outliers were included (r = 0.239; *p* = 0.122) but did correlate when they were excluded (r = 0.324; *p* = 0.034).

With regard to amyloid plaques, there were significant positive correlations with frontal plaque measures both with (r = 0.377; *p* = 0.0126) and without (r = 0.432; *p* = 0.005) serum LPS outliers, in addition to significant positive correlations in total plaque measures both with (r = 0.385; *p* = 0.011) and without (r = 0.486; *p* = 0.001) outliers. Temporal plaque measures missed significance when outliers were included (r = 0.294; *p* = 0.056) but were significant with outliers excluded (r = 0.417; *p* = 0.007). When assessing tau pathology in the form of neurofibrillary tangles, we noted that frontal tangles positively correlated with serum LPS both with (r = 0.350; *p* = 0.022) and without (r = 0.405; *p* = 0.009) outliers. When considering temporal tangles, correlations were not significant in either case. However, measures of total tangles were significant with outliers removed (r = 0.361; *p* = 0.020) but lost significance when outliers were included (r = 0.288; *p* = 0.062). The positive correlations we noted with many of the plaque and tangle pathology measurements did not extend to CAA, whose measures did not correlate significantly with serum LPS regardless of the inclusion or exclusion of the serum LPS outliers.

#### 3.6.3. Brain Tissue LPS Negatively Correlates with CAA, but Not MMSE or Other Pathological Hallmarks

After establishing correlations between serum LPS and various AD hallmarks, we examined whether these correlations also existed between brain tissue LPS measures and these pathologies. We found no correlations between tissue SFG and ITG LPS levels and MMSE scores, Braak scores, or plaque and tangle scores. However, we did find interesting negative correlations between tissue LPS and CAA levels (frontal, temporal, and total), that were notably stronger in the ITG and specifically significant in that tissue but not in SFG tissue (Table 8). We are uncertain why this unique negative association between tissue LPS and CAA exists, as it implies that when CAA is high, LPS is low and vice versa. CAA results from the deposition of amyloid fibrils in the form of amyloid beta (Aβ_1–40_) in the media layer of blood vessels in the brain, primarily small to medium arteries [88]. It is possible that the amyloid accumulation in the vasculature is induced by the presence of microbes attempting to enter the brain, which would be consistent with recent reports that Aβ plays a physiological role as an antimicrobial peptide [14,15,17].

#### 3.6.4. Neither Serum Nor Brain Tissue LPS Levels Correlate with PMI

The PMI could potentially have an effect on LPS levels detected in brain tissue if there was post-mortem leakage of blood-borne LPS into tissues before they could be frozen or otherwise preserved. Across our subject groups, there was no significant difference in the average PMI (range = 2.8 to 3.2 h). We also found no significant correlations between serum LPS levels and the PMI, or between brain tissue LPS levels and the PMI, suggesting that the PMI, particularly the relatively short PMIs of subjects in this study, does not affect LPS measurements (Table 9).

#### 3.6.5. Lack of Correlation of LPS and LTA Measures with Comorbid Peri-Mortem Conditions

As acute or chronic infections present near the time of death might affect LPS and LTA measurements, we looked for correlations between those values in the serum and brain and signs of infectious processes in our subjects based on post-mortem assessments by the BSHRI. Across all associated morbidities identified by the BSHRI (including a variety of infections or inflammatory conditions), there was no significant correlation of serum LPS, with the exceptions of pyelonephritis and pulmonary coccidioidomycosis (Table 10), which is a fungal infection (often persistent) that might affect immune function or become symptomatic in immune-compromised individuals. Similarly, there was generally no association of brain levels of LPS or LTA with the single exception of hepatitis with LTA levels in the SFG. Despite reports of increased blood–brain barrier disruption in individuals carrying APOE4 alleles [89], there was no significant correlation between the number of these alleles and any of our LPS and LTA measures.

## 4. Summary and Conclusions

In summary, we have confirmed that a wide variety of bacterial DNA can be found in brain tissue from aged individuals, regardless of whether AD-associated neuropathology or cognitive decline is present. Furthermore, we identified five major phyla of bacteria that are commonly present in brain tissue and showed that the distribution of those phyla varied distinctly between spatially separated brain regions within the same set of subjects. We validated our sequencing data using measurements of bacterially derived LPS and LTA in brain tissue from those same subjects, showing that the relative amounts of those molecules aligned closely with the relative abundances of Gram-negative versus Gram-positive bacteria identified via sequencing.

One of the unique strengths of this study was the comparison of bacterial DNA signals between two brain regions as well as peripheral tissues from the same set of subjects. In structuring the study in this manner, we answered the question of whether microbial DNA in the brain is distributed in a relatively consistent manner throughout or whether it varies based on local conditions that might be affected by differences in vascular infiltration, glymphatic drainage, and proximity to, or neuronal pathways from, the nasal and oral cavities. Our findings suggest that one of these factors, or perhaps others we have not listed, affects the bacterial taxa that are likely to be found in the different regions as we found the patterns of bacterial taxa were strongly associated with the specific brain region sampled. There are various routes by which infectious agents could arrive in the brain, chief among them being the bloodstream [90,91]. Indeed, the blood–brain barrier is known to be heterogeneous across various brain regions, reflecting the differing functional requirements across brain regions [92]. Recently, it was demonstrated that endothelial cells forming the blood–brain barrier are heterogeneous in different regions of the brain and are altered by aging and in AD [93,94] although the cortex may be relatively less susceptible to age-related changes [94]. Thus, depending on the means by which various microbes interact with the endothelium to access various environments, this could influence where they infiltrate. The structure of the experiments we report here did not allow for a determination of route of entry. Because the microbes identified in the present work comprise an assortment of microbes common to other areas of the body, including both the gastrointestinal and respiratory tracts as well as the skin and the blood, it is possible that they could have arrived in the brain through multiple routes.

A major limitation of this study, as with other low-biomass microbiome studies, was the potential for the contamination of the tissues by exogenous DNA. As described in detail in the methods section, great care was used in the handling of the tissues at all stages of processing in an attempt to mitigate the potential for contamination. Because a major potential for contamination occurs during the acquisition of the tissue post-mortem, the outer surfaces of the tissue samples were removed with sterile/DNA-free scalpels prior to processing. The handling of all tissues was performed inside a biosafety cabinet under sterile conditions at all times. During DNA extraction and enrichment for microbial DNA, microbial DNA-free water was used in all steps involving water. Water blank reagent control samples were run in concert with subject tissue samples through all steps and the resulting sequencing data were compared to identify any potential contaminating sequences. Additional support for our findings that microbes are present in the brain and not simply contaminants introduced during processing comes from our ability to identify high levels of LPS and LTA in the brain via ELISA and the close alignment of those data with our DNA sequencing findings.

While we were not able to link any specific bacterial taxa to the presence of AD or MCI, what we did unequivocally find in this study was a robust microbial signal in the brain tissue in nearly all subjects. Given the nature of this tissue and the fact that it sits behind the blood–brain barrier, these findings would suggest that the brain is not fully isolated from microbes present in the rest of the body. Our findings would imply the presence of a relatively normal brain microbiome, at least in the older individuals included in our study. Since microbiomes in other areas of the body have been found to strongly influence overall health, we feel that it is important to further explore the presence of a potential brain microbiome, which could be important for general brain function and which could be linked to other neurological effects, both favorable and unfavorable.

## Figures and Tables

**Table 1:** Study subject characteristics.

	NDN (*n* = 12)	HPC (*n* = 12)	MCI (*n* = 12)	AD (*n* = 12)
Clinical Information				
Age in years, mean (SD)	79.7 (12.2)	90.3 (5.1)	88.1 (8.6)	81.3 (8.3)
Sex of subjects (male/female)	7/5	6/6	8/4	7/5
Mean post-mortem interval, hrs (SD)	2.93 (1.0)	2.84 (0.9)	3.06 (0.8)	3.2 (0.5)
Mini-mental state examination score, mean (SD)	28.8 (0.8) ^a^	27.6 (1.4)	26.0 (3.4) ^b^	10.3 (7.6)
APOE alleles: number of subjects with each genotype 2/3, 2/4, 3/3, 3/4	0, 0, 8, 4	2, 0, 7, 3	0, 1, 9, 2	0, 0, 6, 6
Neuropathological Features				
Plaque and tangle scores (# subjects scoring none, sparse, moderate, or frequent). Plaque scores based on CERAD.				
Frontal plaque score	10, 2, 0, 0	0, 0, 1, 11	1, 1, 1, 9	0, 0, 0, 12
Temporal plaque score	10, 2, 0, 0	0, 0, 1, 11	2, 1, 1, 8	0, 0, 0, 12
Frontal tangle score	11, 0, 0, 0 ^b^	9, 3, 0, 0	5, 6, 1, 0	0, 2, 1, 9
Temporal tangle score	10, 2, 0, 0	2, 7, 3, 0	2, 5, 3, 2	0, 1, 0, 11
Braak staging (# subjects stage I to stage VI)	5, 3, 4, 0, 0, 0	0, 1, 2, 9, 0, 0	0, 1, 1, 9, 1, 0	0, 0, 0, 0, 6, 6

Abbreviations: NDN = Nondemented Normal Controls; HPC = High-Pathology Controls; MCI = Mild Cognitive Impairment; AD = Alzheimer’s disease; SD = standard deviation. ^a^ Scores not available for two subjects. ^b^ Score not available for one subject.

**Table 2:** ANOSIM and PERMANOVA results by phyla.

	ANOSIM		PERMANOVA	
Paired Testing	*p*-Value	r-Value	*p*-Value	F-Value
ITG vs. SFG	0.0020	0.0835	0.0002	9.7336
Neuropathology vs. Non	0.0310	0.0979	0.3177	1.0857
Cognitive decline vs. Non	0.4720	−0.0014	0.5181	0.7550

**Table 3:** PERMANOVA results comparing phyla by region and group.

Phylum	*p*-Value	F-Value
Bacterial phylum by Region (SFG, ITG)		
Actinobacteria	0.0682	3.4004
Bacteroidetes	0.0537	3.8337
Cyanobacteria	0.2444	1.4309
Firmicutes	0.0020	10.2892
Proteobacteria	0.0001	20.2925
Bacterial phylum by Subject Group (AD, MCI, HPC, NDN)		
Actinobacteria	0.3779	1.0484
Bacteroidetes	0.0792	2.3102
Cyanobacteria	0.6997	0.5401
Firmicutes	0.2958	1.2654
Proteobacteria	0.9388	0.1422

**Table 4:** E(ρ) values for correlations between Phyla.

Phylum	Actinobacteria	Bacteroidetes	Cyanobacteria	Firmicutes	Proteobacteria
Actinobacteria	1	−0.26798052	−0.32736980	−0.31092280	0.07942094
Bacteroidetes		1	−0.34221850	−0.31132280	−0.06330904
Cyanobacteria			1	−0.24166700	−0.24249271
Firmicutes				1	−0.25641202
Proteobacteria					1

**Table 5:** ANOSIM and PERMANOVA results by orders of Proteobacteria.

	ANOSIM		PERMANOVA	
Paired Testing	*p*-Value	r-Value	*p*-Value	F-Value
ITG vs. SFG	0.0010	0.1310	0.0034	6.3156
Neuropathology vs. Non	0.6560	−0.0203	0.6441	0.4950
Cognitive decline vs. Non	0.7150	−0.0088	0.4396	0.8567

**Table 6:** Correlation analyses of serum LPS versus SFG and ITG tissue LPS and of serum LPS versus subject age.

	Serum LPS including Outliers		Serum LPS with Outliers Removed	
Paired Testing	Correlation	*p*-Value	Correlation	*p*-Value
Serum LPS vs. SFG LPS	−0.0632	0.6945	−0.0231	0.8831
Serum LPS vs. ITG LPS	−0.2174	0.1721	−0.1484	0.3423
Serum LPS vs. Age	−0.0859	0.5932	−0.0218	0.5520

**Table 7:** Correlation analyses of serum LPS versus MMSE score and AD-associated pathologies including Braak score, plaques, tangles, and CAA.

	Serum LPS including Outliers		Serum LPS with Outliers Removed	
Pathology	Correlation	*p*-Value	Correlation	*p*-Value
MMSE	−0.3735	0.0176	−0.4350	0.0063
Braak score	0.2395	0.1219	0.3238	0.0389
Frontal plaque	0.3774	0.0126	0.4324	0.0048
Temporal plaque	0.2935	0.0561	0.4173	0.0066
Total plaque	0.3851	0.0108	0.4856	0.0013
Frontal tangle	0.3498	0.0215	0.4047	0.0087
Temporal tangle	0.1827	0.2409	0.2821	0.0739
Total tangle	0.2876	0.0615	0.3611	0.0204
Frontal CAA	0.0327	0.8351	0.1248	0.4368
Temporal CAA	0.0311	0.8431	0.1174	0.4647
Total CAA	0.1436	0.3581	0.2680	0.0902

**Table 8:** Correlation analyses of CAA versus brain tissue LPS levels.

	SFG LPS		ITG LPS	
Pathology	Correlation	*p*-Value	Correlation	*p*-Value
Frontal CAA	−0.1569	0.2870	−0.2892	0.0462
Temporal CAA	−0.1223	0.4075	−0.4393	0.0018
Total CAA	−0.2015	0.1696	−0.4335	0.0021

**Table 9:** Correlation analyses of post-mortem interval (PMI) versus LPS in serum and brain tissue.

Paired Testing	Correlation	*p*-Value
PMI vs. Serum LPS (+) outliers	−0.0218	0.8895
PMI vs. Serum LPS (−) outliers	−0.0526	0.7441
PMI vs. SFG LPS	0.1630	0.2682
PMI vs. ITG LPS	0.2613	0.0728

**Table 10:** Correlation analyses of comorbid peri-mortem conditions with LPS and LTA.

Peri-Mortem Condition	N	Serum LPS (*p*-Value)	SFG LPS (*p*-Value)	SFG LTA (*p*-Value)	ITG LPS (*p*-Value)
Acute pneumonia	19	0.5943	0.364	0.7206	0.5225
Acute/chronic pyelonephritis	5	0.0099	0.4491	0.6719	0.3163
Acute/chronic pancreatitis	2	0.8805	0.4638	0.5535	0.9778
Pulmonary coccidioidomycosis	5	0.0332	0.9183	0.4480	0.4491
Acute/chronic bronchitis	2	0.0610	0.1932	0.5717	0.9778
Acute/chronic cystitis	9	0.5047	0.4285	0.5063	0.7647
Acute/chronic thyroiditis	6	0.7599	0.8612	0.2338	0.1078
Acute/chronic gastritis	7	0.7599	0.9523	0.6464	0.8109
Acute/chronic esophagitis	4	0.0671	0.2292	0.0613	0.9850
Acute/chronic duodenitis	2	0.5854	0.8522	0.9999	0.7729
Acute/chronic hepatitis	5	0.6259	0.4084	0.0218	0.4285
Acute/chronic cholecystitis	2	0.3512	0.7729	0.2360	0.1565
Chronic sialadentis	2	0.9292	0.6327	0.3845	0.7122
Other possible infections or inflammatory conditions	9	0.2477	0.8421	0.6752	0.9246
Other conditions predisposing to infection or inflammation	25	0.7842	0.3683	0.0963	0.332

## Data Availability

Sequence reads and the associated R code for Section 2.9 are freely available via figshare at the following doi:10.6084/m9.figshare.25902031. All raw sequencing data files (fastq) have been uploaded to the NCBI Sequence Read Archive (SRA) and assigned BioProject accession number PRJNA1129202 (assigned on 28 June 2024. Following publication, these files can be accessed via the following link: https://www.ncbi.nlm.nih.gov/sra/PRJNA1129202).
